# Harnessing Bacteriophages to Combat Antibiotic-Resistant Infections in Africa: A Comprehensive Review

**DOI:** 10.3390/antibiotics13090795

**Published:** 2024-08-23

**Authors:** Kafayath Fabiyi, Kevin Sintondji, Jerrold Agbankpe, Phenix Assogba, Hornel Koudokpon, Boris Lègba, Elodie Gbotche, Lamine Baba-Moussa, Victorien Dougnon

**Affiliations:** 1 Research Unit in Applied Microbiology and Pharmacology of Natural Substances, Research Laboratory in Applied Biology, Polytechnic School of Abomey-Calavi, University of Abomey-Calavi, 01, Cotonou P.O. Box 2009, Benin; kafayath.fabiyi@uac.bj (K.F.); kevin.sintondji@uac.bj (K.S.); agbankpe.jerrold@urmapha-epacuac.bj (J.A.); phenix.assogba@urmapha-epacuac.bj (P.A.); hornel.koudokpon@epac.uac.bj (H.K.); boris.legba@urmapha-epacuac.bj (B.L.); gbotchee@gmail.com (E.G.); 2 Laboratory of Biology and Molecular Typing in Microbiology, Faculty of Sciences and Techniques, University of Abomey-Calavi, Cotonou P.O. Box 1604, Benin; laminesaid@yahoo.fr

**Keywords:** bacteriophage therapy, multidrug-resistant pathogens, phage characterization, clinical applications of phages

## Abstract

The conventional treatment of bacterial infections with antibiotics is becoming increasingly ineffective due to the emergence of multidrug-resistant (MDR) pathogens. This literature review explores the potential of bacteriophages as an alternative or adjunctive therapy to antibiotics in combating MDR infections in Africa. This analysis focuses on current research regarding the integration of phage therapy into African healthcare, highlighting its challenges and opportunities. This review begins with the AMR crisis and the need for new treatments, then covers the history, mechanisms, benefits, and limitations of phage therapy. Key African studies are summarized, identifying major obstacles such as regulatory issues, infrastructure, and research standardization. Research efforts in West Africa that have made notable progress in bacteriophage research are highlighted. This review concludes with recommendations for policymakers, researchers, and healthcare professionals to enhance the development and use of phage therapy in Africa, aiming to reduce antibiotic resistance and improve patient outcomes. By addressing the identified challenges and leveraging the unique advantages of phages, there is potential to significantly mitigate the impact of antibiotic resistance and improve patient outcomes in Africa.

## 1. Introduction

The rapid spread of antimicrobial resistance (AMR) is a major global challenge and a key priority for several leading organizations including the World Health Organization (WHO), the European Centre for Disease Prevention and Control (ECDC), and the National Institute for Health Research (NIHR) [1]. AMR has been exacerbated by the decline in the discovery of new antibiotics, the persistent use of antibiotics, and consequently, the rapid emergence of bacterial strains resistant to existing and new antibiotics [1]. The excessive, uncoordinated, and unjustified use of these antimicrobial molecules in human and veterinary medicine and in agriculture has led to widespread AMR in all sectors and calls for a One Health approach [2]. The situation is alarming in countries with limited resources where infectious diseases, poverty, and malnutrition are endemic [3].

The most clinically relevant multidrug-resistant (MDR) pathogens are collectively known as ESKAPE (*Enterococcus faecium*, *Staphylococcus aureus*, *Klebsiella pneumoniae*, *Acinetobacter baumannii*, *Pseudomonas aeruginosa*, *Enterobacter* species) [4]. These bacteria are responsible for the majority of infectious diseases. According to the WHO, more than 700,000 people worldwide die every year from antibiotic-resistant infections and this figure could rise to 10 million a year by 2050 without concerted action [5]. The emergence and spread of antibiotic resistance expose patients to an increased risk of therapeutic failure [6]. The priority pathogens identified by WHO in 2017, including ESKAPE pathogens, were responsible for 929,000 AMR deaths and 3.57 million AMR-related deaths in 2019.

Faced with the growing threat of AMR and the decline in the discovery of last-resort antibiotics, it is more important than ever to look for alternative solutions. Several avenues are being explored, including the research and development of new antimicrobial molecules based on medicinal plants (natural substances), antimicrobial peptides, probiotic lactic acid bacteria, and bacteriophages. One of the most promising of these is the therapeutic application of lytic bacteriophages (phages), which are viruses that kill bacteria. Phage therapy has a long history of use in countries such as Georgia, Poland, and France [7], where it has been used as an adjunct to or replacement for antibiotics to treat bacterial infections for over 100 years. It is crucial to widen access to this therapy either as an alternative or as a complement to antibiotic treatment. If phage therapy is to be developed and made accessible to all, it is advisable to focus on bacterial diseases for which no other treatment exists and those with high levels of AMR [8].

Indeed, phages were discovered almost a century ago and their antibacterial potential was recognized shortly afterwards. In fact, the first trials of phage therapy preceded Fleming’s discovery of penicillin by around ten years [9]. Phage therapy is the use of phages to treat infectious diseases of bacterial origin [10]. It is a strategy based on the lytic cycle of virulent phages. This type of treatment is only used as a last resort in human medicine in North America and Europe. While the use of bacteriophages as antibacterial agents is not a universal solution, it is realistic in economic and temporal terms, with temporary adverse reactions or symptoms. Bacteriophages are the most abundant and diverse biological entities on the planet. In aquatic environments, phages play a major role in biogeochemical cycles [11].

In recent years, the use of phages in Africa to biologically control multi-resistant bacteria has also increased as this antimicrobial therapy does not appear to be associated with chemical residues or toxicity. Studies have been carried out in Côte d’Ivoire, Egypt, Ethiopia, Ghana, Kenya, Nigeria, South Africa, Tunisia, and Uganda on the biocontrol of phages in agriculture, animal husbandry, veterinary medicine, and human health [12,13,14,15,16,17]. These studies have demonstrated the lytic activity of phages in inhibiting bacterial proliferation in vivo. Research into phages is still in its infancy in African countries, particularly in West Africa, due to several challenges, including limited funding and resources, a lack of specialized infrastructure, underdeveloped regulatory frameworks, and a shortage of trained professionals. Moreover, bacteriophages are highly specific and variable biological entities. Their efficacy can depend on many factors, including environmental conditions, types of host bacteria, and the genetic characteristics of the phages themselves. This variability makes it difficult to standardize phage research, particularly in less advanced countries with very limited resources, where rigorous consistency is needed to obtain reliable and comparable results.

In this context, it is therefore important to take stock of the various research efforts on the continent in order to provide a comprehensive scientific basis for researchers wishing to engage in phage therapy research with a view to combating antimicrobial resistance and controlling multidrug-resistant infections. It is with this in mind that this literature review has been initiated; its main aim is to examine the challenges and opportunities associated with integrating phage therapy into the fight against antibiotic-resistant infections in Africa. This review will explore current research initiatives on the continent and discuss the obstacles to be overcome in order to make phage therapy a viable clinical reality in Africa.

## 2. Results 

### 2.1. Analysis of Primary Data 

This literature review is the result of data collection in various databases. A total of 80 articles were identified. After an initial selection, duplicate articles were eliminated. We therefore retained 38 articles. A thorough reading of the articles and compliance with the inclusion and exclusion criteria led to a second selection of 14 articles for this study. 

### 2.2. Current State of Bacteriophage Biocontrol of Bacteria in West Africa

Several projects on bacteriophages have been launched in Africa. The data in Table 1 presents studies about bacteriophage research in West Africa. According to Table 1, there is growing interest in phage research, but most research is focused on the isolation and characterization of phages and the evaluation of their lytic activity. 

More than half of the research on biocontrol and phage therapy against multidrug-resistant ESKAPEE bacteria conducted in West Africa has focused on *Escherichia coli* phages, with strains mainly producing Shiga-like toxins (STEC) (Table 1). After *E. coli* phages, studies on *Pseudomonas aeruginosa* phages came second, followed by studies on *Klebsiella pneumoniae*, *Acinetobacter baumanii*, and *Staphylococcus aureus* phages. The findings also highlight significant strides in bacteriophage research across West Africa, with notable studies conducted between 2016 and 2024. In Benin, researchers isolated and characterized three novel *Acinetobacter baumannii* phages from hospital wastewater, contributing to the *Autographiviridae* family [18]. In Nigeria, various studies explored phages from effluent water, soil, sewage, and surface water, targeting multiple bacteria such as *Escherichia coli*, *Bacillus* spp., *Klebsiella pneumoniae*, and *Pseudomonas aeruginosa*, with findings that include the first bacteriophages from Tropical Africa and characterization of lytic phages against *E. coli* O157 [19,20,21,22,23,24]. Additionally, studies in the Ivory Coast investigated phages infecting *E. coli* and *Enterobacter cloacae* in wastewater and novel phages from rodents with lytic activity on clinical *Enterobacteriaceae* strains [25,26]. Ghana and Senegal also made contributions, with research on phage applications for controlling Shiga toxin-producing *E. coli* in food and the complete genome sequences of *Klebsiella pneumoniae* phages from Dakar, respectively [15,27]. Most of the studies reviewed showed that various phages had a high level of efficacy against the pathogens listed by the WHO. It should be noted that all the studies included in this review concerned the isolation, characterization, and genomic analysis of phages and the evaluation of their efficacy in inhibiting MDR bacteria responsible for various infections in humans and animals. All the articles studied dealt with in vitro biocontrol. This aspect of phage research is the most important, as it demonstrates the effectiveness of phages in treating human and animal infections. However, there are still gaps in phage product design and development, as well as in preclinical and clinical phage trials for the development of phage therapy in Africa. 

### 2.3. Current State of Bacteriophage Biocontrol of Bacteria in the Rest of the African Continent

The African continent has seen a steady increase in research initiatives focused on the application of phage therapy to combat antibiotic-resistant bacterial infections. The studies reviewed span a diverse range of countries, including Côte d’Ivoire, Egypt, Ethiopia, Ghana, Kenya, Nigeria, South Africa, Tunisia, and Uganda. These studies have demonstrated the lytic activity of phages in inhibiting bacterial proliferation in various settings, including agriculture, animal husbandry, veterinary medicine, and human health [12,13,14,15,16,17].

One notable study conducted in Nigeria evaluated the efficacy of bacteriophages isolated from sewage in treating MDR *E. coli* infections in poultry [15]. The study found that phage therapy significantly reduced bacterial counts and improved survival rates in infected birds. Similarly, a study in South Africa demonstrated the potential of phages to control MDR *Staphylococcus aureus* in dairy cows, highlighting the broader applicability of phage therapy in veterinary settings [17].

### 2.4. Challenges in Implementing Phage Therapy in Africa

Despite the promising results, several challenges impede the widespread adoption of phage therapy in Africa. These challenges are summarized below.

#### 2.4.1. Research and Training Challenges

The understanding of phage therapy and biocontrol is limited in Africa. There is a lack of personnel trained in phage isolation, characterization and production [28]. Phages are relatively large particles, with an average size of 100–200 nm, which require specific approval processes and adapted production and purification techniques, such as specialized chromatography and filtration [10].

There are many challenges in phage therapy research and clinical trials. One of the main challenges is the heterogeneity of the target bacteria and the need to develop phage cocktails specific to these bacteria. Indeed, pathogenic bacteria vary considerably in terms of strains, serotypes and genetic resistance. Bacteria can express different phenotypes depending on their environment and stage of growth. For example, bacterial biofilms are often more resistant to treatments because of the protective extracellular matrix. Phages need to be specifically selected for their ability to infect and lyse resistant bacterial strains, which requires rigorous preclinical testing and more complex selection processes [29]. The lack of clinical trials for the few phage cocktails formulated is also a handicap to the development of phage therapy in Africa. Many clinical trials are hampered by difficulties in recruiting participants, often due to mistrust of new therapies, exacerbated by a lack of information about phage therapy. Another challenge is the lack of standardization in phage isolation protocols, phage standardization, environmental variability, phage production, purification, and variability in phage preparations. This could affect the reproducibility and comparability of clinical trial results. Variability in phage formulations and storage conditions may also influence the therapeutic efficacy of phages [30]. The process of isolating bacteriophages requires specialized laboratory equipment and specific technical skills. In the less developed countries of West Africa, particularly Benin, there are few or no laboratories with the facilities needed to carry out advanced phage research. In addition, limited access to reagents and culture materials makes phage isolation more complex. Natural environments, such as wastewater and soil, are rich reservoirs of phages due to human activities, antibiotic residues present in these ecosystems, and common practices. As a result, the isolation of bacteriophages from these sources requires rigorous techniques to ensure the purity and efficacy of the phages isolated. This presents a real challenge in terms of equipment, reagents, consumables, and skilled personnel. In addition, phage characterization requires equipment such as electron microscopes, which most African countries do not have. The lack of funding, support, or partnerships is also a major obstacle limiting research into phage therapy. In addition, facilities for the large-scale production of phage-based medicines are lacking, necessitating the establishment of regulatory frameworks to ensure the safety, efficacy, and quality of phage-based products [10]. Variability in phage formulations and storage conditions can also influence the therapeutic efficacy of phages. Finally, collaboration between phage therapy researchers and research institutes must form the fundamental basis for research into bacteriophages and the development of phage therapy in West Africa. The first case of phage therapy in the United States, which led to the creation of the Center for Innovative Phage Applications and Therapeutics (IPATH), took place when the University of California San Diego Medical Center had neither a phage bank nor a dedicated phage laboratory. This success was made possible by multi-institutional cooperation, in particular with Texas A&M University, and mainly by personal initiatives explaining the clinical need to use phages [31].

#### 2.4.2. Institutional and Socio-Economic Challenges

Phage therapy is only officially approved in some Eastern countries such as Poland, Georgia and Russia, and its use remains very limited in Africa [32]. More clinical trials are needed to demonstrate the safety and efficacy of phage therapy and biocontrol in Africa, particularly in the treatment of multidrug-resistant bacterial infections [33].

The economic challenges associated with phage therapy are particularly significant in West Africa. The cost of research, clinical trials, and the infrastructure needed to produce phages on a large scale is often prohibitive. National policies in West African countries provide very little funding for research [34]. Very few countries see research and innovation as the basis for sustainable and equitable development. This lack of priority for research translates into a lack of adequate resources to support innovative projects such as phage therapy [35]. Phage production requires specialized facilities and trained personnel, which represents a significant investment in terms of initial costs and maintenance [36]. In addition, the cost of researching and developing new phage cocktails is high due to the numerous safety and efficacy tests required before a product can be accepted for preclinical and clinical testing and ultimately marketed. Each stage of this process is costly and requires stable, long-term funding [31]. Another major obstacle is the cold chain required to preserve phages. Phage therapy requires storage at specific temperatures to maintain the efficacy of the phages, which in turn requires the mobilization of considerable energy resources. In many West African countries, the energy infrastructure is precarious and unreliable, making it even more difficult to set up efficient cold chains [37]. This not only increases operational costs, but also poses significant logistical challenges [38]. Finally, the lack of investment in healthcare and research infrastructures limits the capacity to develop and implement phage therapy [34]. Existing infrastructures are often insufficient to support large-scale, complex research projects. Consequently, even when external funding is obtained, the lack of adequate infrastructure can prevent the effective implementation of phage therapy projects.

#### 2.4.3. Socio-Cultural Challenges

Socio-cultural perceptions play a crucial role in the acceptance of phage therapy in West Africa. In many communities, there is a strong preference for conventional antibiotic treatments, fueled by traditional medical practices and a mistrust of innovative therapies. This preference is often reinforced by the fact that antibiotics are well-established, widely available, and have a history of successful use. In contrast, phage therapy, although promising, is still largely unknown or poorly understood by many, which can lead to a reluctance to adopt it [39]. Another major obstacle is the low level of awareness of phage therapy among healthcare professionals and patients. Many healthcare professionals are not sufficiently informed about the potential benefits of phages and may be skeptical about their efficacy [40]. This situation is exacerbated by the lack of training on new therapies in medical education programs, which limits the spread of knowledge about phage therapy.

Phage therapy also faces stigmas associated with the use of viruses as therapeutic agents. The term ‘virus’ often has a negative connotation, evoking diseases and infections rather than therapeutic solutions [41]. This perception can hinder the acceptance of these treatments, despite their potential. To overcome this barrier, it is crucial to run targeted awareness and education campaigns to demystify phage therapy and highlight its benefits [42]. In addition, cultural differences and traditional beliefs can influence the perception and acceptance of innovative medical treatments, including phage therapy. For example, in some cultures, natural treatments and traditional remedies are preferred to modern medical interventions. To promote acceptance of phage therapy, it is essential to engage local communities and opinion leaders to raise awareness and educate about the benefits of phages. The involvement of traditional and religious leaders can be particularly effective in changing perceptions and encouraging the adoption of new treatments [43]. Finally, efforts must be made to adapt awareness-raising messages to the specific cultural contexts of different communities. Appropriate communication initiatives, testimonials from patients who have benefited from phage therapy, and collaboration with the local media can help to build a better understanding and wider acceptance of this therapy [44].

As a summary, the key challenges are as follows:

1. Regulatory hurdles: The lack of clear regulatory frameworks for phage therapy in many African countries poses a significant barrier. This makes it difficult to conduct clinical trials and obtain approval for phage-based treatments [45].
2. Infrastructure limitations: Limited laboratory infrastructure and resources hinder the isolation, characterization, and production of bacteriophages. This challenge is particularly pronounced in resource-poor settings [36].
3. Standardization issues: The high specificity and variability of bacteriophages require standardized protocols for their use in therapy. However, the lack of standardized research methodologies and quality control measures complicates the development and implementation of phage therapy [46].
4. Public awareness and acceptance: There is a general lack of awareness and understanding of phage therapy among healthcare providers and the public. This can lead to resistance to adopting phage therapy as a viable treatment option [47].
5. Funding constraints: Limited funding for research and development of phage therapy is a major obstacle. Most research initiatives are reliant on external funding, which can be inconsistent and insufficient [48].

### 2.5. Opportunities for Integrating Phage Therapy

Despite these challenges, there are significant opportunities for integrating phage therapy into the healthcare systems of African countries. To effectively promote the development of phage therapy in Africa, it is crucial to invest in basic and applied phage research [40]. This includes financial and institutional support for the isolation, characterization and banking of phages adapted to pathogens prevalent in Africa, such as *Escherichia coli*, *Klebsiella pneumoniae*, *Pseudomonas aeruginosa*, and *Acinetobacter baumanii* [32]. These efforts should also include training African researchers in the molecular biology and biotechnology techniques needed to manipulate and exploit phages. Establishing collaborations between African researchers and international institutions can facilitate knowledge transfer and capacity building [48]. Networks such as the African Phage Forum can play a crucial role in promoting phage research and therapy.

Secondly, it is essential to communicate research results effectively to enable rapid knowledge sharing and interdisciplinary collaboration. This will foster a common understanding of the challenges and opportunities associated with phage therapy, encouraging innovative approaches and strategic partnerships between researchers, clinicians, and the pharmaceutical industry [44]. Creation research networks and data-sharing platforms can also accelerate the development of phage-based solutions adapted to African contexts. Advocacy efforts to develop and implement regulatory frameworks for phage therapy are essential. Engaging policymakers and regulatory bodies can help create an enabling environment for clinical trials and the use of phage-based treatments. Also, raising awareness and providing training for healthcare providers on the benefits and applications of phage therapy can enhance its acceptance and implementation. Educational campaigns targeting the public can also help demystify phage therapy and promote its use [44]. Leveraging innovative research approaches such as metagenomics and synthetic biology can enhance the isolation and characterization of bacteriophages [49]. These technologies can help identify novel phages with broad-spectrum activity against MDR pathogens. Finally, to integrate phage therapy into African clinical practice, it is imperative to conduct rigorous, well-controlled clinical trials to assess the efficacy, safety, and economic benefits of this therapeutic approach [50]. These trials should be designed in collaboration with local and international health authorities, taking into account the epidemiological and clinical specificities of the regions. The results of these studies will serve as a basis for developing clinical guidelines and regulatory policies adapted to the use of phages in the treatment of antibiotic-resistant infections in Africa. Implementing pilot programs to demonstrate the efficacy and safety of phage therapy in treating specific bacterial infections can provide valuable evidence and build confidence in its use [36]. Successful pilot programs can pave the way for larger-scale clinical trials and broader adoption of phage therapy.

## 3. Methods

### 3.1. Search Method and Selection Criteria

The Web of Science (WoS), PubMed, Scopus, Cochrane Library, Google Scholar, and Embase databases were searched independently by two authors until March 2024. Publications were searched using various combinations of the following terms: “Antibiotic resistance AND Africa”, “Bacteriophagy AND multidrug-resistant infections AND Africa”, “Phage therapy AND clinical trials AND Africa”, “Phage therapy AND public health initiatives AND Africa”, “Challenges of phage therapy AND combating antibiotic resistance AND Africa”, “Antimicrobial resistance AND integration of bacteriophages AND Africa”, and “Applications of phage therapy AND treatment of infections”. The reference lists of selected articles were also manually reviewed and appropriate articles were included. Abstracts of papers published at scientific meetings or other events were not reviewed as they did not provide sufficient detail for quality assessment. The titles and abstracts of all articles were reviewed by one reviewer and the eligibility of reviewed articles was assessed by two independent investigators on the basis of the following criteria.

### 3.2. Inclusion Criteria

The inclusion criteria were as follows: (i) studies published in peer-reviewed journals, (ii) studies written in English, and (iii) studies investigating phage therapy as a treatment for bacterial infections and its application in Africa.

### 3.3. Exclusion Criteria

The exclusion criteria were as follows: (i) reviews that did not focus on Africa, (ii) studies of therapeutic agents other than phages, and (iii) studies not related to bacterial infections.

### 3.4. Screening and Selection of Studies

A two-step process was followed. Initially, the titles and abstracts of all identified studies were screened to exclude clearly irrelevant studies. For those that appeared to meet the inclusion criteria, the full text was obtained and further reviewed for final inclusion. Any discrepancies between the reviewers regarding the inclusion of particular studies were resolved through discussion and consensus.

### 3.5. Data Extraction

Data were extracted from selected studies using a predesigned data extraction form. The following information was collected: study characteristics (authors, year of publication, country, and setting), type of study (laboratory-based, clinical trial, case report, etc.), target bacteria, types of phages used, method of phage isolation and characterization, outcomes of phage therapy, and any reported challenges or limitations.

## 4. Conclusions

This review highlights the significant progress in bacteriophage research across West Africa from 2016 to 2024, emphasizing their potential in fighting bacterial infections and reducing antimicrobial resistance. Researchers in Benin, Nigeria, Ivory Coast, Ghana, and Senegal have successfully isolated and characterized phages from various environments such as hospital wastewater, effluent water, soil, and sewage. These phages target a wide range of bacteria, including *Acinetobacter baumannii*, *Escherichia coli*, *Bacillus* spp., *Klebsiella pneumoniae*, and *Pseudomonas aeruginosa*, demonstrating their usefulness for both treatment and biocontrol. The findings show that West Africa is making valuable contributions to phage research, which could lead to new solutions for public health challenges related to antimicrobial resistance. Continued support for phage research in Africa is crucial for developing these innovative approaches further.

## Figures and Tables

**Table 1:** Studies about bacteriophage research in West Africa.

N°	Countries	Years	Samples	Bacteria Host for Phage	Title of the study	Phage Family	Authors
1.	Benin	2023	Hospital wastewater	*Acinetobacter baumannii*	Isolation and characterization of three novel *Acinetobacter baumannii* phages from Beninese hospital wastewater	*Autographiviridae*	[18]
2.		2022	Sewage and surface water	*Bacillus* spp., *Escherichia coli*, *Klebsiella pneumoniae*, *Pseudomonas aeruginosa*, *Staphylococcus aureus, and Xanthomonas* sp.	Nigerian phages: The first bacteriophages from Tropical Africa	Myoviridae, Siphoviridae or Podoviridae	[19]
3.			Effluent water	*Escherichia coli* O157:H7	Identification of *Escherichia coli* O157:H7, Characterization, and Host Range Analysis of Bacteriophages Infecting Some Selected Pathogenic Bacteria from Jos, Nigeria	-	[20]
4.		2023	Poultry	*ESBL-E. coli*	Antibiotic resistance genes, mobile elements, virulence genes, and phages in cultivated ESBL-producing Escherichia coli of poultry origin in Kwara State, North Central Nigeria	Myoviridae Siphoviridae Inoviridae and Podoviridae	[21]
5.		2022	Wastewater	*Pseudomonas aeruginosa*	Isolation of *Pseudomonas aeruginosa* Phages from Wastewaters	-	[22]
6.		2022	Wastewater	Shiga toxin-producing E. coli O157:H7	Isolation of *Escherichia coli* phages from wastewater	-	[23]
7.	Ivory Coast	2021	Wastewater	*E. coli*, *Enterobacter cloacae*	Investigation of phages infecting *E. coli* and *Enterobacter cloacae* in sewage	-	[12]
8.		2017	Water and fishes	*P. aeruginosa*, *Enterobacter aerogenes*, *K. pneumoniae, and E. coli*	New identification of phages and their application for multidrug strains in Abidjan	-	[24]
9.		2020	Water	*P. aeruginosa*	Biocontrol of multidrug-resistant *P. aeruginosa* infection by phages in aquaculture	-	[25]
10.		2020	Rodent intestines	*E. coli*	First novel phages from rodents with lytic activity on clinical *Enterobacterales* strains	*Myoviridae*	[26]
11.		2023	Lake water	*E. coli, P. aeruginosa, and E. cloacae*	Multiple adaptations of bacteriophages in Lagoon Ebrie: Indicators of permanence microbiological pollution in the environment, Ivory Coast, West Africa	-	[24]
12.	Ghana	2020	Irrigation ponds and streams	Shiga toxin-producing *E. coli*	Application of phage cocktail for control of shiga toxin-producing *E. coli* in foods and food contact surfaces	-	[15]
13.	Senegal	2024	Sewage water	*Klebsiella pneumoniae*	Complete genome sequences of two *Klebsiella pneumoniae* phages from Dakar	-	[27]

## Data Availability

All data generated and/or analyzed during the current study are included in this published article. The datasets used and/or analyzed during this study are also available from the corresponding author on reasonable request.
